# The Rollercoaster of Research: A Day in the Life of Yuwei Jiang, University of Illinois Chicago, USA

**DOI:** 10.1002/cph4.70166

**Published:** 2026-05-10

**Authors:** Paul Trevorrow, Yuwei Jiang

**Affiliations:** ^1^ John Wiley and Sons Ltd. Bognor Regis UK; ^2^ University of Illinois Chicago Chicago Illinois USA

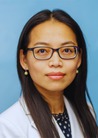

## 1 Significant Contributions to the Field

Yuwei's most significant contributions to the field so far center on adipogenesis—specifically, how fat cells are generated from stem and progenitor cells, how their identities are established, and how their development is regulated.

A major focus of her and her team's work has been understanding the crosstalk between adipose progenitor cells and their surrounding niche. Adipose tissue is not made up of fat cells alone; it also includes immune cells, vascular cells, and other supporting cell types. How these different cell populations communicate with one another is essential for forming and maintaining healthy adipose tissue. Understanding this cellular communication has been a key theme of their research.

In their previous studies, Yuwei's group identified specific markers for adipose progenitor cells, which allow researchers to target and study them more precisely. They have also begun to uncover why these progenitor cells sometimes fail to generate functional fat cells. In particular, the team has established important connections between progenitor cells and immune cells and shown how their signaling interactions change—or fail—in conditions such as aging and obesity. These findings have helped clarify why adipose tissue function declines in metabolic disease. Rather than a complete loss of progenitor cells, the issue often lies in disrupted communication within the tissue microenvironment.

In addition to these contributions, over the past decade, work from Yuwei's lab has advanced our understanding of beige fat cell origins and the mechanisms that control their formation.

## 2 A Day in the Life of…

Yuwei's days have settled into a quieter rhythm than they once had. With one child already in college and the other preparing to go, the constant pull of parenting has softened. Mornings are calmer now—still full, but more her own.

She usually wakes up alongside her dog, who insists on being the first priority of the day. That early walk is brief but grounding, a small pause before work takes over. Soon after, Yuwei heads straight to the lab. By the time she arrives, her mind has already shifted into research mode.

The first task of the day is always email. She reads everything carefully, making sure nothing urgent slips through. Some messages can be answered immediately; others require more thought, more time, and get flagged to return to later. Staying on top of communication is essential—students, collaborators, administrators, and journals all rely on timely responses. Before the morning really begins, Yuwei makes sure her inbox is under control. Teaching does take up part of her schedule, but not so much anymore.

When she does get stretches of uninterrupted time, Yuwei spends a significant portion of it writing. Writing grants, reviewing grants. Writing papers, reviewing manuscripts. Depending on the week, writing can take up anywhere from a quarter to half of her working time. It's demanding, often mentally exhausting, but it's also where ideas take shape and research moves forward.

These days, Yuwei rarely runs experiments herself. Instead, she oversees the work of her lab. Once a week, she sets aside half a day—4 or 5 h—in the animal room. Her lab does extensive mouse work, and maintaining healthy, well‐managed colonies is critical. She checks in with lab members, makes sure protocols are being followed, and keeps an eye on colony size. Space and resources are limited, and overexpansion can become costly, so careful planning matters. It's hands‐on in a different way than before, but no less important.

Relaxation, for Yuwei, often means rest. On weekends, she allows herself to sleep in and catch up on rest she doesn't get during the week. Grocery shopping has become a surprisingly calming ritual—practical, slow, and disconnected from work.

She's trying to exercise more, though it's a work in progress. Long hours at her desk have made movement easy to neglect. Still, she makes an effort. On some weekends, she meets friends to play pickleball, a welcome break from research and responsibilities. It keeps her active, social, and firmly grounded in life outside the lab.

## 3 Current Research

In Yuwei's lab, they focus on understanding the development of fat cells—the cells many of us tend not to like. Early in her career, she became particularly interested in where these cells come from. By understanding their cellular origins and the regulatory mechanisms that control them, researchers can begin to think about how to increase or reduce fat cell numbers in a targeted way.

This is important because fat cells are actually essential for maintaining metabolic health. In conditions such as obesity, diabetes, and many age‐related metabolic diseases, adipose tissue plays a critical role. Understanding how fat cells develop, where they originate, and how they are regulated is therefore central to many important health‐related questions.

Since establishing her own lab, Yuwei has continued to follow these questions, but with a focus on a specific type of fat cell known as beige fat cells. These are distinct from white fat cells and are related to, but different from, brown fat cells. Beige fat cells are inducible, meaning that under certain conditions, white adipose tissue can be converted into beige adipose tissue.

What makes beige fat cells particularly interesting is that they function like brown fat: instead of storing energy, they burn it. This ability to burn glucose and fat is especially beneficial in the context of obesity and diabetes. In an ideal world, we could use our own fat tissue as a way to combat excess fat—letting the body's natural machinery do the work.

However, a major challenge is that although beige fat cells are beneficial, their number and activity decline in both aging and obesity. This has been observed in animal models as well as in human studies. Much of Yuwei and her team's current research focuses on understanding why this decline occurs and whether it is possible to restore beige fat cells to improve metabolic health in obese or aging populations.

Yuwei's work, along with studies from other groups, suggests that the progenitor cells that give rise to beige fat are still present. The problem is not necessarily that these cells are lost, but that they become dysfunctional. They lose the ability to differentiate into beige fat cells, likely due to factors such as cellular aging, stress, inflammation, or disrupted communication with their surrounding environment.

This realization is exciting because it means the system is still there—it just isn't working properly. Several of our studies suggest that it may be possible to rejuvenate these aged, dysfunctional progenitor cells. By targeting specific pathways, including hormonal signals and metabolites derived from the gut microbiota, we can restore their function.

In mouse models, Yuwei and her team have found that even in obese or aging conditions, restoring progenitor cell function allows animals to produce beige fat again and maintain better metabolic health. These findings are very encouraging, and they are now working to better understand the underlying mechanisms and explore the potential therapeutic applications of this research in more advanced models. Ultimately, the goal of this work is to translate these biological insights into strategies that improve metabolic health and combat obesity‐associated diseases.

## Funding

The authors have nothing to report.

## Conflicts of Interest

The authors declare no conflicts of interest.

## Data Availability

The authors have nothing to report.

